# Unraveling reaction-diffusion-like dynamics in urban congestion propagation: Insights from a large-scale road network

**DOI:** 10.1038/s41598-020-61486-1

**Published:** 2020-03-17

**Authors:** Leonardo Bellocchi, Nikolas Geroliminis

**Affiliations:** Urban Transport Systems Laboratory (LUTS) École Polytechnique Fédérale de Lausanne (EPFL), GC C2 390, Station 18, Lausanne, CH-1015 Switzerland

**Keywords:** Civil engineering, Complex networks

## Abstract

We study the dynamical process of congestion formation for large-scale urban networks by exploring a unique dataset of taxi movements in a megacity. We develop a dynamic model based on a reaction and a diffusion term that properly reproduces the cascade phenomena of traffic. The interaction of these two terms brings the values of the speeds on road network in self-organized patterns and it reveals an elegant physical law that reproduces the dynamics of congestion with very few parameters. The results presented show a promising match with an available real data set of link speeds estimated from more than 40 millions of GPS coordinates per day of about 20,000 taxis in Shenzhen, China.

## Introduction

Traffic models are fundamental for engineers and researchers in transportation to design and apply control strategies in order to mitigate urban congestion. Combining realistic modeling of congestion and efficient traffic control for large scale urban systems remains a big challenge. This is due to the high unpredictability of choices of travelers (in terms of route, time of departure and mode of travel), the uncertainty in their reactions to the actions of others (users or controllers), the spatiotemporal propagation of congestion, the lack of coordinated actions coupled with the limited infrastructure available^1,2^ and the strong scatter of the data for link-level traffic sensors. The growing research interest on this topic is testified also by several recent works, (for example^3–11^) where the authors try to recover travelers’ behavior through extensive data-driven trip prediction, i.e. collecting GPS of mobile phones. While there is a vast literature on congestion dynamics, control and spreading in one-dimensional traffic systems with a single mode of traffic^12–17^, most of the analysis at the network level is based on simplistic models or detailed simulation, which requires a large number of input parameters (sometimes not observable with existing data) and cannot be solved in real-time.

In literature, we can distinguish four families of traffic flow models: network, macroscopic, mesoscopic and microscopic models, that often are implemented in micro-simulators (a survey on this argument can be found in ref. ^18^). Based on the purpose of the research, one can choose to engage a model of an appropriate scale. Network level models normally use a regional description of the network and aggregate traffic variables. The advantages of this type of models are the low computation cost and the possibility to use well-defined empirical relations between the main traffic variables: flow, density and speed, known as Fundamental Diagram (FD). Note that these relations are not for individual links as in the traditional link-level models, but for homogeneously congested regions of a city. It has been empirically observed^19,20^ that by spatially aggregating the highly scattered plots of flow vs. density from individual links (e.g. 1 min data), the scatter decreases and a well-defined curve exists between space-mean flow and density. Thus instead of high scatter local FDs, a Macroscopic Fundamental Diagram (MFD) is established at a network level^21–23^. The shape of MFD is a property of the network infrastructure and control and not very sensitive to the demand, but it depends on the spatial heterogeneity of congestion. This is important network property, as details at the micro-level are not necessary to describe congestion propagation. It can also be utilized to introduce simple control strategies to improve mobility in multi-region city centers building on the concept of an MFD, like in refs. ^24,25^, and others. Recent findings from empirical and simulated data^26,27^ have identified the spatial distribution of vehicle density in the network as one of the key components that influence the shape and the scatter of an MFD. These findings are of great importance because the concept of an MFD can be applied for heterogeneously loaded cities with multiple centers of congestion if these cities can be partitioned into a number of homogeneous spatially connected clusters^19,28–30^.

The mesoscale models simulate traffic for smaller network elements, like roads or for groups of cars. Another powerful and detailed class of models are those based on the micro-simulators. They are agent-based models where the decision variables, necessary to run microsimulations, are chosen at the driver level. This fact implies a high computational cost and also greater stochasticity of the results. The car-following model is one of the most common for lower level modeling, proposed in ref. ^31^ and in a simplified version in ref. ^32^. Nevertheless, it emerges, for example in ref. ^33^, the complexity to calibrate the huge amount of parameters (route and mode choice, traffic dynamics, demand generation and more) in the widely used micro-simulators for driving behavior models and the difficulty to find a universal and definitive values system.

The model proposed here is a link-based model, but without requiring detailed origin-destination information, like turning ratios at intersections, or queue lengths and drivers’ behaviour. The objective of this model is not to predict accurately the speed at link level, but to reproduce network characteristics such as speed distribution, the spatial pattern of congested areas in the road network as well as the relation between mean and standard deviation in quasi-congested period and their evolution over time. In the same spirit as the network MFD approach, it only requires a few parameters to be calibrated. The average link speed (in a time horizon of a few minutes) is the main variable of interest. Nevertheless, mesoscopic models often require tedious calibration as specifying capacity for each link and run route choice algorithms, detailed or aggregated^34–36^.

This work has been inspired by a very general version of the reaction-diffusion phenomenon that is at the basis of various and widespread aspects in nature (described in continuous space in refs. ^37,38^ and generalized for networks in ref. ^39^). As in ref. ^40^, we combine the traffic variables in a dynamical system governed by classical physical laws. In fact, these systems develop self-organized spatial patterns that share, in particular configurations, similar characteristics with those that can be observed during a peak hour for connected components of congested links in urban networks (for example in ref. ^41^). Other works, as in ref. ^42^, point out the influential distance and correlation between the origin of traffic jams and the consecutive spatio-temporal spreading over all the road network during the peak-hours.

The original idea is to invert the common point of view of the models, that is not to estimate congestion given an OD matrix, drivers’ behavior, road capacity, and other specific traffic variables, but look at the product of all these variegate factors as an unique cause that makes the speed values on the road network tractable with a physical dynamical system. Now, it is worth to remark the reasons for which we chose the link-speed as the main variable and not, like in many other classical conservation-based traffic models, the link density. Many real datasets and the most used on-line measurement techniques are nowadays based on probe traffic data as GPS signals from taxis, buses and mobile phone of private cars’ drivers and all of these provide only trustworthy speed information by recording position and time. This technology is considered by far much cheaper and potentially ubiquitous information than the sensors used to measure the density, based mainly on measurements from loop detectors that yield only point density and not link density. For example, in the Shenzhen data used in the paper, the penetration rate is less than 1%, meaning that on average we observe 1 or 2 vehicles every 5 minutes in a link. This information is sufficient to have a proxy of speed, but it creates significant uncertainty if density needs to be estimated.

The more visible advantage of this model with respect to the other models in the literature is that it can reproduce congestion propagation in large-scale networks with thousands of roads by clustering only a few parameters. The dynamical process of urban traffic in networks^43^ is here simulated with an elegant model and without any exogenous information like origin-destination (OD) matrix and drivers’ path choice. Moreover, the distribution characteristics such as standard deviation of link speeds and the average mean are accurately reproduced by the reaction-diffusion model at all moments.

## A reaction-diffusion model

The master differential equation of the proposed model is composed of two parts, a diffusion and a reaction term, operating in a spatial network, represented by a graph ${\mathscr{G}}(N,E)$ of $N$ nodes and $E\subset N\times N$ links.

The diffusion part is regulated by the combinatorial Laplacian $L=A-D$ where $a$ is the corresponding adjacency matrix of graph ${\mathscr{G}}$ and $D$ the diagonal matrix $\{{d}_{ii}={k}_{i}\}$ with ${k}_{i}$ the corresponding node degree of each node $i\in N$. For the purpose of this work, it will be simpler and useful to define the roads of the transportation network as nodes and the intersection points between road segments as links. In particular, the element ${A}_{ij}=1$ if and only if road $i\in N$ and road $j\in N$ are adjacent, $0$ otherwise. The elements on the diagonal of the matrix $L$, ${l}_{ii}=-\,{k}_{i}$, represent the number of the adjacent links of each intersection point $i\in N$. In literature, one might find another definition of the combinatorial Laplacian, with opposite sign: $-1$, instead of $1$, if there is a link between $i$ and $j$, and ${k}_{i}$ in the diagonal. We chose to keep the definition in the cited seminal paper about reaction-diffusion system in networks^39^. The reaction-diffusion equation that we propose was inspired by Fick’s law of concentration of particles, but models with mass and flow conservation have been studied in transportation with various limitations, mostly related to transient states (high scatter FDs) and route choice behavior. We aim to demonstrate that the abstraction effort that consists to pass from a road density description to a link velocity perspective is still valid and that it is worth considering.

For our traffic model the vectorial variable ${\bf{u}}(t)={\{{u}_{i}(t)\}}_{i\in N}$ will represent the vector of average link speed for each road $i$ at a certain time $t$. If then, on the one hand, the diffusion simulates the linear propagation of congestion, on the other hand, the reaction adds to the system a non-linear effect, characteristic of the vehicular traffic, as the non-uniform demand in space and/or in time. In particular, the reaction term will be represented by non-linear function ${f}_{a}(\rho ,{\bf{u}}(t),\widehat{\bar{u}}(t),i,t)$ depending, in general, on a weight $\rho$, on the speeds’ vector ${\bf{u}}(t)$, the expected network average speed $\widehat{\bar{u}}(t)$, on the location $i$ and time $t$ and parameterized by a factor $a$ that represents the reaction rigidity as we will explain in the next section. In the following, we will refer to $f$ as the *reaction function*. The differential equation at the core of the model, at link level $i\in N$, is:

1 $$\frac{d{u}_{i}(t)}{dt}={f}_{a}(\rho ,{\bf{u}}(t),\widehat{\bar{u}}(t),i,t)+\mathop{\sum }\limits_{j=1}^{N}{\sigma }_{ij}{L}_{ij}{u}_{j}(t)+{\varepsilon }_{b},$$

where the reaction and diffusion parameters, $\rho$ and $\sigma$ respectively, can locally depend on space.

The diffusion term ${\sum }_{j=1}^{N}{\sigma }_{ij}{L}_{ij}{u}_{j}(t)$ changes the distribution of link speed values ${u}_{i}$ among the network while the reaction term ${f}_{a}(\rho ,{\bf{u}}(t),\widehat{\bar{u}}(t),i,t)$ is the responsible for the change of the network average speed ($\widehat{\bar{u}}(t)=\frac{{\sum }_{i=1}^{N}{u}_{i}(t)}{N}$). The weights for the effects of two terms can be regulated by their respective parameters $\rho$ and $\sigma$. Finally, ${\varepsilon }_{b}$ is a random number from a zero-mean symmetric density probability function. We can assume that the noise does not influence the network average speed but it actually has an effect on the distribution of the speed values and it measures the ‘randomness’ of the real system. Moreover, the term ${\varepsilon }_{b}$ is related with the estimation of the metastability of the model (similarly to the technique in ref. ^44^) and especially at the optimal configuration of the parameters $({a}^{\ast },{b}^{\ast })$, in the sense that the error remains small (minimal) for limited random input values $-{b}^{\ast } < {\varepsilon }_{{b}^{\ast }} < {b}^{\ast }$. For our applications we considered a uniform distribution for ${\varepsilon }_{b}\in {\mathscr{U}}(-b,b)$. Gaussian white noise ${\varepsilon }_{b}^{G}\in {\mathscr{N}}(0,b/\sqrt{3})$ for the random term, with the same standard variation ($b$/$\sqrt{3}$) has been tested in the model and the results appear totally similar with respect to the uniform distribution.

### Principles of reaction and diffusion

We decided to keep the mathematical form as elegant as possible and look at what are the two fundamental principles that regulate the observable traffic propagation: the linear effect of spatial diffusion and the non-linear interaction between congested and non-congested adjacent links.

The two principles of reaction and diffusion that are expressed in the differential Eq. (1) can be seen as the counterpart of two following empirical facts (observed, also, in ref. ^45^):

1. A congested link leads the drivers to prefer one of its neighbor links;
2. If a link is surrounded by congested links with high probability it will get congested with a brief delay.

In other words, $(P1)$ captures in an rough way the awareness of drivers’ about the traffic condition ahead^46–48^ while $(P2)$ the dispersal/aggregation dynamics that occur in an emerging congested pattern during the onset and offset of peak-hour events^49^.

For each link $$i$$ and time $$t$$, assertion $$(P1)$$ is simulated by the diffusion term $${\sum }_{j=1}^{N}{\sigma }_{ij}{L}_{ij}{u}_{j}(t)={\sum }_{j\in {N}^{i}}{\sigma }_{ij}$$$$({u}_{j}(t)-{u}_{i}(t))$$, where $${N}^{i}$$ is the set of the adjacent link to $$i$$. When diffusion is applied, the consequence is a value transfer from a link $$i$$ to its neighbors $$j\in {N}^{i}$$, that means to get higher or lower value $${u}_{i}$$ proportionally to the difference with its adjacent links maintaining the sum of all link speed values unchanged. On the other hand, $$(P2)$$ is made effective by the reaction term. After computing the difference in speed between a link $$i$$ and its neighbors, the reaction function and the parameter $$a$$ calibrate the increment or decrement of the value $${u}_{i}$$ at each time step $$dt$$. For this reason, for each link $$i$$, we introduced as argument of the reaction function $$f$$ the variable $$\Delta {u}_{i}(t)={\sum }_{j=1}^{N}{\rho }_{ij}{A}_{ij}({u}_{j}(t)-{u}_{i}(t))={\sum }_{j\in {N}^{i}}{\rho }_{ij}({u}_{j}(t)-{u}_{i}(t))$$. It is worthy to remark here that the non-linearity of $$f$$ and the lack of an explicit counterbalancing term in the Eq. (1) distinguishes the reaction from the diffusion, that is the simulation of the effect of an exogenous demand (characterized by $$\widehat{\bar{u}}(t)$$, weighted by $$a$$ and locally influenced by $$\Delta {u}_{i}(t)$$) from the endogenous diffusion dynamics. The assumption $$(P2)$$ restricts the type of reaction function that we can choose. In particular, it has to be monotonic in $$\Delta {u}_{i}$$ in order to follow the trend of the values of the neighborhood, bounded and non linear, to be able to translate the complexity of the congestion phenomenon and its characteristic self-dependent and self-enhanced growth and dispersion^50,51^.

### Parameters region-to-region

An urban transportation network is composed by streets classifiable according to their characteristics (speed limit, capacity, number of lanes, etc.) and functionality (e.g. type of intersection control) as periphery, primary or secondary roads, highway and others. Therefore, it is reasonable to imagine that the traffic flow between two roads belonging to two different types can be correlated in a different way than, for example, two consecutive links.

In this work, we apply another differentiation using clustering algorithms that divide the street network into large zones based on the level of congestion or well defined Macroscopic Fundamental Diagrams (for example, as done in refs. ^29,52^). For this reason, in order to reach more accuracy, it can be useful to set reaction ($\rho$) and diffusion ($\sigma$) parameters for each cluster. For example, traffic congestion might propagate differently in freeways than urban streets. In general, $\rho$ and $\sigma$ will be represented by two squared matrices of dimension $R$, where $R$ is the number of the regions or types identified in the network. We will denote ${{\mathscr{N}}}_{r}$ the set of links belonging to a certain region $r$. Using this notation, the parameters used in Eq. (1) will be ${\sigma }_{{R}_{i}{R}_{j}}$ and ${\rho }_{{R}_{i}{R}_{j}}$ with ${R}_{i},{R}_{j}\le R$ respectively the index of the clusters of road $i$ and road $j$.

The values of these parameters have been calibrated looking at the principal effect that they have on the speed distribution. In particular, the reaction parameter $\rho$ influences the changes of the network average speed and the diffusion parameter $\sigma$ affects the distribution of the link speed values, namely on the standard deviation. It is a fact that a higher value for $\sigma$ leads the network speed distribution towards more homogeneity.

## Results

In the case that congestion grows in a network, e.g. during the morning onset of congestion, the parameters of the model do not significantly change and the value of the reaction term $a$, that can be set at the beginning of the peak hour, is proportional to the average decrements of the network speed. But, in order to simulate the fluctuating traffic behavior during the day, instead of using demand information in the model (the rate of new trips that are generated), which is difficult to obtain, we integrate an adaptive mechanism based on real-time observable measurements. More specifically, we monitor every some time interval $\tau$ (e.g. 20 minutes) the online error between the real $\widehat{\bar{u}}(t)$ and the estimated network average speed $\bar{u}(t)$. For the whole-day case, we looked for a symmetric function, bounded and where the online adaptation algorithm is easy to apply and set up. In fact, the on-line adaptation is based on a constant ${\alpha }_{{t}_{k}}=a* (\widehat{\bar{u}}({t}_{k})-\bar{u}({t}_{k}))$ that brings the system to increase or decrease the network average speed based on the real online measurements $\widehat{\bar{u}}({t}_{k})\left(=\frac{{\sum }_{i\in N}{\hat{u}}_{i}({t}_{k})}{N}\right)$ at time ${t}_{k}$, with $k=1,\ldots ,K$, where $k$ is the total number of the calls of the adaptive algorithm. For this aim, we chose to use the function ${f}_{a}(\rho ,{\bf{u}}(t),\widehat{\bar{u}}(t),i,t)=tanh({\alpha }_{t}+\Delta {u}_{i}(t))$, with ${\alpha }_{t}={\alpha }_{{t}_{k}},\forall {t}_{k}\le t < {t}_{k}+\tau ={t}_{k+1}$, that respects all our requirements. We want to notice the other functions, as the sigmoid or *arctan*, could fit the principles that we sustained above, but the main scope of this work is to demonstrate the existence of physical dynamics of traffic propagation described by a local differential equation of type (1).

### Adaptive parameters implementation: a whole day example

**Figure 1:**
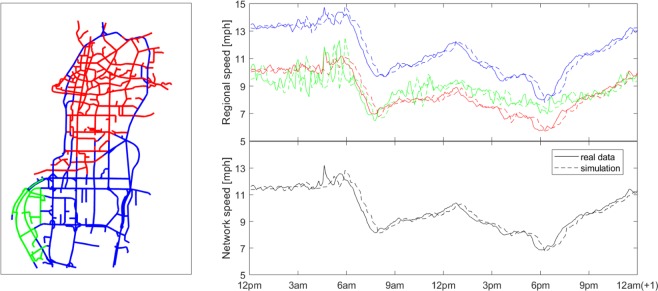
Simulation accuracy for regional average link speeds. In the panel on the top-right, it is reported the average speed for each cluster of Shenzhen downtown. The continuous lines correspond to the real data while the dashed line come from the simulation of the reaction-diffusion model with $a=0.29$, $b=1.2$ and $K=70$. Each color corresponds to the region of the same color on the Shenzhen map on the right. On the bottom, the average speed for all the network. The simulation is referred to the whole day on ${7}^{th}$ September 2011 and the 3 regions are the result of an off-line clustering algorithm that tends to minimize the variance inside each region of the city in congested scenario.

**Figure 2:**
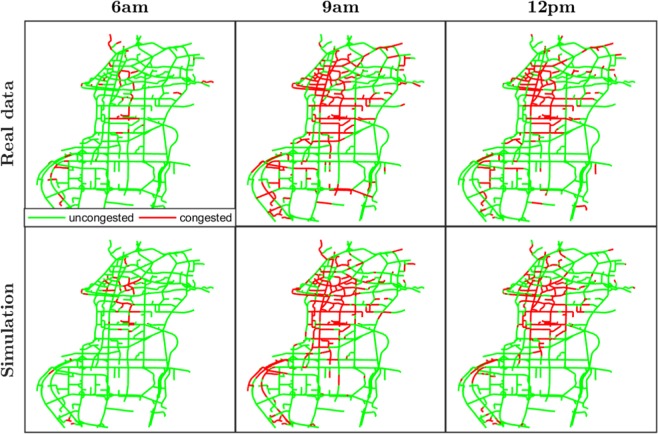
Map of congested links from real data and simulation during morning peak-hour. Links colored in red are considered congested (velocity less than 8 [mph]) at 6 am, 9 am and 12 pm of Day 1.

**Figure 3:**
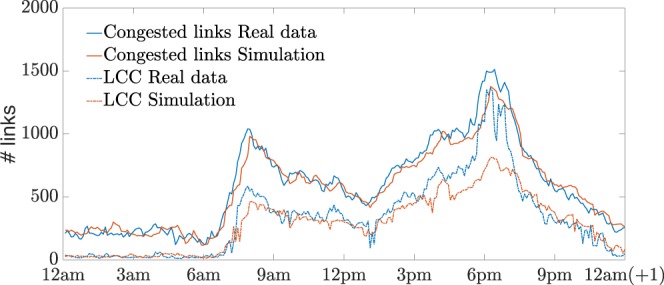
Comparison between the LCC of congested links in real data and in the simulation of the model. The continuous lines indicate the total number of the links (out of 2013) that are considered congested at each time $t$ of the day, that is with a velocity less than 8 [mph]. The blue color is used for the real data and the red for the simulation. The dashed lines report the size of the corresponding LCC.

**Figure 4:**
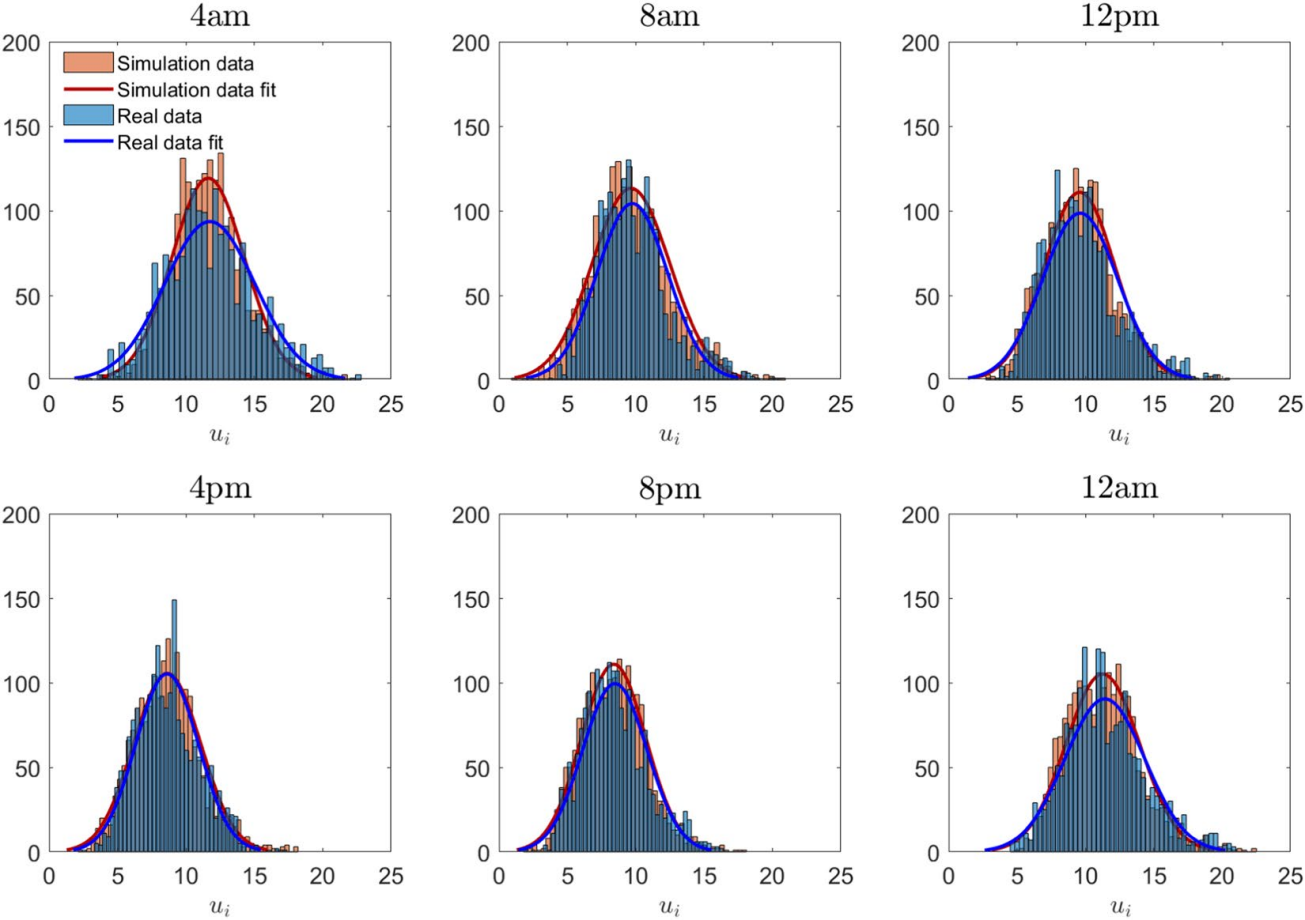
Histogram of the link speeds ${\{{u}_{i}\}}_{i\in N}$ distribution. The panels from the top-right to the bottom-left report every 4 hours the histograms of the link speeds distribution on Shenzhen network for the real data of Day 1 (in blue) and the corresponding simulation (in red). Being $F(x,t)$ and $G(x,t)$ the two empirical cumulative density functions (ECDFs) of the observed and the simulated link speed distribution at time $t$, the two-sample Kolmogorov-Smirnov (KS) statistic $D(t)={\max }_{x}| F(x,t)-G(x,t)|$ has an average of $\widetilde{D}=0.04$ overall the day. In this picture, all the distributions reported from 8am to midnight pass the KS test with $\alpha =5 \%$, while it gives negative answer during the night (2 am–6:40 am) due to the scarcity of GPS data in that time window. For an easier visual comparison, the fitted normal distribution of the two histograms have been represented in each panel.

We tested the reaction-diffusion model to simulate a whole day with on- and off-peak of the congestion in the Shenzhen downtown. In particular, all the results that we show in Figs. 1–4 are relative to the ${7}^{th}$ September 2011 (Day 1), after the calibration process from all the available dataset explained in the corresponding section.

For each region $r$, we define ${\widehat{\bar{u}}}^{r}(t)=\frac{1}{| {{\mathscr{N}}}_{r}| }{\sum }_{i\in {{\mathscr{N}}}_{r}}{{\hat{u}}}_{i}(t)$ and ${\bar{u}}^{r}(t)=\frac{1}{| {{\mathcal{N}}}_{r}| }{\sum }_{i\in {{\mathcal{N}}}_{r}}{u}_{i}(t)$ the average speed at time $t$ across the links ${{\mathscr{N}}}_{r}$ belonging to $r$ for the real data and the simulation respectively. As we anticipated in the previous section, we considered for this scope the symmetric function ${f}_{a}(\rho ,{\bf{u}}(t),{\widehat{\bar{u}}}^{r}(t),i,t)=tanh({\alpha }_{{t}_{k}}^{r}+\Delta {u}_{i}(t))$, for each region and time ${t}_{k}\ \le \ t < {t}_{k+1}$, with ${\alpha }_{{t}_{k}}^{r}$ to add to the argument of the reaction function in order to calibrate the model. With the control parameter ${\alpha }_{t}^{r}=a\ast ({\widehat{\bar{u}}}^{r}(t)-{\bar{u}}^{r}(t))$, we adjust the reaction function in order to follow each regional speed ${\bar{u}}^{r}(t)$ and, as consequence, the whole network speed $\bar{u}$ (Fig. 1). The weight $a$ is a scalar to regulate the strength of the adaptive algorithm and has been chosen to be the same for all regions. Note that $a$ can be estimated in real-time as it only looks for the difference of regional speeds and, in addition, this is also straightforward to estimate if data from loop detectors is available in line with the MFD theory^19^. In all the results shown in this paper, the parameter ${\alpha }_{k}^{r}$ has been evenly updated 70 times in a day ($k=1,2,\ldots ,K=70$), that corresponds to every 20 minutes in a 24h day.

We define a connected component of congested links as a set of links whose observed velocity is below a certain threshold, that we fixed at 8 [mph], and connected each other by at least a path all contained in the same set. The value of 8 [mph] has been chosen in accordance with the traffic flow theory of MFD, where the definition of congestion is when the saturation flow is reached and any increment in road density corresponds to a decrease of traffic flow. Experience and large-scale measurements from different case-studies, as in ref. ^19^, demonstrate the consistence of the choice of this particular value. In Fig. 2, we illustrate as example the evolution of the spatial pattern of the congestion during the morning peak-hour while in Fig. 3 we analyze the fluctuation of the Largest Connected Component (LCC) of congested links during the whole day simulation and we compared it with the real data. As one can notice, the model reproduces the size of the largest component very precisely except for the evening peak hour where the model underestimates it. Nevertheless, the total number of the congested links in all the network is reproduced with high accuracy during the whole simulation. We can observe the typical congestion pattern formation during a peak hour period: from some critical links the congestion spreads in neighbor links forming large connected components (a network analysis of this transition mechanism is done in refs. ^42,45,53^). This phenomenon is also enhanced by the gridlock effect and traffic spillback^2,54^. Applications of percolation theory to traffic jams have attracted attention recently and provide intuitive explanations for the identification of critical links and bottlenecks in a network^55,56^. Usually, a link is considered not operational when the velocity is very low and it is removed from a connected cluster. Nevertheless, in the physical world of traffic congestion, when a link reaches such a state, it contains a large number of vehicles that still need to travel to their destinations. Thus, these links (even at a very congested state) are still connected with the other parts of the network. Reaction-diffusion models still consider the whole network during speed propagation and might be considered an alternative to study these phenomena.

We want to stress the fact that during the simulation we never upload the link speeds of the model with the available real data but we only consider the average over a macro-region (or the whole network) to update the parameter ${\alpha }_{t}$ for the argument of the reaction function. In fact, the model reproduces by itself the local dynamics given the general traffic condition and this guarantees the continuity and consistency of the simulated speed values and the applicability of the model using the estimations for macro-region deducible from historical data and easier online measurements. In this sense, the model is able to simulate the on-peak and off-peak of the congestion without losing the accuracy on the distribution of the link speed values (Fig. 4) and the evolving spatial congestion pattern.

### Calibration of adaptive strength $a$ and randomness $b$

In order to calibrate the parameters $a$ and $b$ of the model for the case of the whole day simulation, we used the measure

2 $$MS=\frac{1}{K}\mathop{\sum }\limits_{k=1}^{K}\sqrt{{(\bar{u}({t}_{k})-\widehat{\bar{u}}({t}_{k}))}^{2}+{(std({\bf{u}}({t}_{k}))-std(\widehat{{\bf{u}}}({t}_{k})))}^{2}}$$

where, as mentioned before, $K$ is the number of the calls of the adaptive algorithm, ${t}_{k}$ the time at which each call $k$ corresponds and $std(\,\cdot \,)$ stands for standard deviation of the speed distribution vector. The quantity $MS$ is the average Euclidean distance in the plane between each corresponding couple of points $(Meanvalue,Standarddeviation)$ of the estimated speed ${\bf{u}}(t)$ and real speed data $\widehat{{\bf{u}}}(t)$. For each value $a=0.11,0.12,\ldots ,0.4$, we change the interval $[-b,b]$ ($b=0,0.1,\ldots ,2.9$) relative to the noise ${\varepsilon }_{b}$ and compute the accuracy in the relation between network average speed and standard deviation of the link speed distribution through the quantity defined in formula (2).

**Figure 5:**
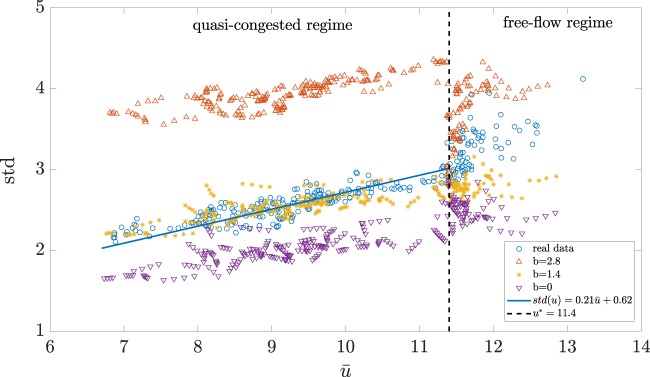
Relation between the mean and the standard deviation of the distribution of link speeds (Day 1). Every 5 minutes the values of network average speed $\bar{u}(t)$ and standard deviation $std({\bf{u}}(t))$ ($t=1,2\ldots ,288$) are reported in this graph and visualized as circled points. In particular, the blue circled points correspond to the real data measurements and they are compared with the simulations for 3 different levels of noise ${\varepsilon }_{b}$, that is $b=0$ (no noise), $b=1.2$ (best-fit) and $b=2.8$. The parameter $a$ has been fixed at $0.29$. The parameter ${\alpha }_{k}^{r}$ has been evenly updated every 20 minutes ($K=70$). In the quasi-congested regime the values of the linear relation between standard deviation and network speed (continuous blue line) are $\Gamma =0.21$ and $\lambda =0.62$.

**Figure 6:**
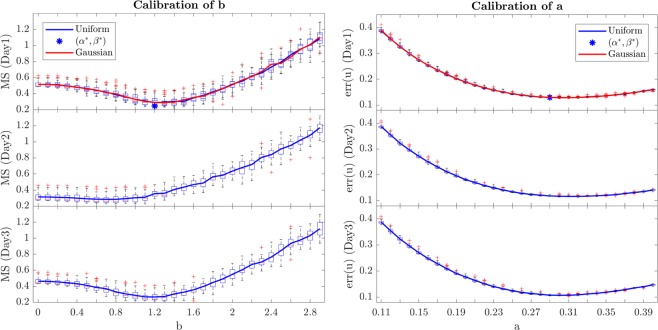
Calibration of a and b parameters for 3 days. For each value $a=0.11,0.12,\ldots ,0.40$, we run 30 simulations by increasing the noise parameter $b$ from $0$ to $2.9$ by steps of $0.1$. In the panel on the left, it is shown the obtained distribution of MS defined in formula (2), when the adaptation is made every 20 minutes in a day. On the right, for each $a$, it is reported the boxplot of the distribution of the standard deviation of the error between the real data $\widehat{\bar{u}}(t)$ and the simulation $\bar{u}(t)$, $err(u)=\sqrt{\frac{1}{T}{\sum }_{t=1}^{T}{(\widehat{\bar{u}}(t)-\bar{u}(t))}^{2}}$. For Day 1, we also show the same results considering a normal distribution of the noise ${\varepsilon }_{b}^{G}$ instead of the uniform presented in the main text. In each plot, we put in evidence the average of the error values, in particular, in blue for the uniform distribution of ${\varepsilon }_{b}$ and in red for the Gaussian distribution of ${\varepsilon }_{b}^{G}$. Both for the $err(u)$ and for the $MS$, the uniform and the Gaussian noise distribution ${\varepsilon }_{b}$ gives equivalent results.

For the calibration of $a$ and $b$, we used the data from different working days in September in Shenzhen. We report here 3 representative cases that we named Day 1, 2 and 3. We notice that the best parameters for the model are almost the same for all days. In order to minimize the error among all dataset, a unique couple $({a}^{\ast },{b}^{\ast })=(0.29,1.2)$ has been fixed and applied for all days and the standard deviation of the link speeds remains always less than 0.13 [mph] and the MS error below 0.3. The results showed in this section all refer to Day 1 (${7}^{th}$ September 2011) if not specified otherwise. Figure 5 shows the relation between standard deviation and average of the link speed values for 3 iconic values of $b$ when $K=70$. It clearly appears a mathematical relation between these variables that the reaction-diffusion model reproduces and maintains all simulation long, especially when the congestion starts to appear. We call this the *quasi-congested period*, when the network speed $\bar{u}$ is below the critical threshold ${u}_{qc}^{\ast }=11.4$ [mph]. The mathematical reason of the value of ${u}_{qc}^{\ast }$ has been discussed in the following section about heteroscedasticity. In this regime, we observed that the standard deviation $std({\bf{u}})$ decreases as the congestion in the network increases (and speed decreases) and the relation with the network speed $\bar{u}$ goes from a scattered behaviour ($\bar{u}\ \ge \ {u}_{qc}^{\ast }$) to a well-defined linear dependence ($\bar{u} < {u}_{qc}^{* }$). Empirically, the standard deviation of link speed distribution can be approximated by a linear relation in terms of the mean speed $\bar{u}$, that is $std({\bf{u}})=\Gamma \bar{u}+\lambda$. This shows that even complex and large urban networks experience a simple law between the level of congestion (as expressed by a space-mean speed) and spatial heterogeneity (as expressed by standard deviation of link speeds distribution) when speed is below a critical value. We found that this relation is maintained also for different days, as show in the following. For the points in the quasi-congested period ($\bar{u}\ \le \ {u}_{qc}^{\ast }$) out of the 288 measurements par day evenly sampled from our 72-hours link speed data, the best linear fitting is reached with $\Gamma =0.21$ and $\lambda =0.62$. Figure 5 shows also how that only $\lambda$ depends on $b$ while the slope $\Gamma$ remains the same at fixed $a=0.29$. Figure 6 represents the boxplot of the average error in mean speed for each parameter $a$ varying the noise ${\varepsilon }_{b}$. All the panels in Fig. 6 show the existence of an optimum value for $a$ and $b$ that varies just a little in different days. We observe that this relation between average mean speed and standard deviation reaches its best fit in $b=1.2$ for Day 1 and 3 and in $b=0.7$ for Day 2 where the MS error defined in formula (2) has its minimum value. However, it seems that Day 2 does not show a clear U-shape like the other two days. In the panel on the right, it is reported the obtained distribution of the standard deviation error $err(u)=\sqrt{\frac{1}{T}{\sum }_{t=1}^{T}{(\widehat{\bar{u}}(t)-\bar{u}(t))}^{2}}$ between the real data $\widehat{\bar{u}}(t)$ and the simulation $\bar{u}(t)$ when the adaptation is made every 20 minutes in a day. For Day 1, we also show the same results considering a normal distribution of the noise ${\varepsilon }_{b}^{G}$ instead of the uniform ${\varepsilon }_{b}$ presented in the text. In order to compare the two distributions, we considered a normal distribution with zero-mean and same standard deviation than the uniform one (that in our case is $2b$/$\sqrt{12}$).

### Heteroscedasticity in link speed distribution

**Figure 7:**
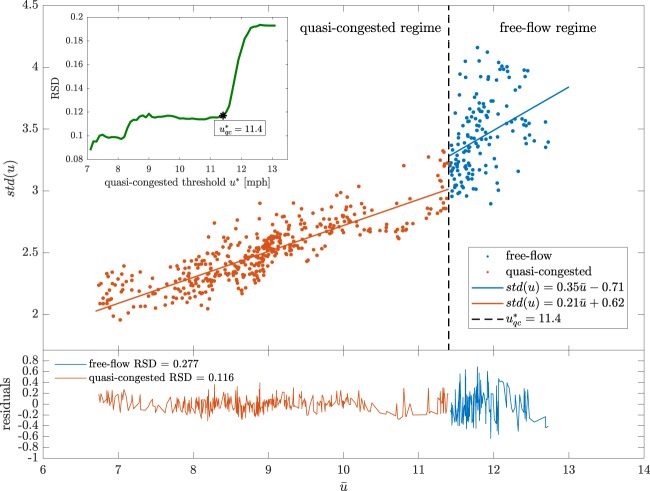
Heteroscedasticity in the distribution of link speed values. The data plotted here are from 3 days of the dataset of Shenzhen and one point every 20 minutes. From this scatter plot it is possible to deduce that the relation between average link speeds and standard deviation is independent of the day and it follows two distinguished regimes: the free-flow and the quasi-congested one. The subpanel in the top-left represents the Residual Standard Deviations (RSD), defined as the average norm of the residual of a linear fitting of the data whose average speed $\bar{u}$ is less than the threshold ${u}^{\ast }\in [7,13]$. At ${u}^{\ast }=11.4$ it clearly appears a critical increment of data dispersion and, consequentially, the heteroscedasticity. Therefore, the value ${u}^{\ast }=11.4$ has been used to distinguish the two regimes. In the plot on the bottom, it is reported the residual plot of the linear regression of the points of the flee-flow part (blue) and the quasi-congested part (red). The RSD for these two parts are 0.277 and 0.116 respectively.

Merging the data of Day 1, 2 and 3 as explanatory representatives of the dataset, we have been able to show the implicit and fundamental relation already investigated in Fig. 5. We can clearly distinguish two regimes for the standard deviation/average speed relation by two factors: the slope and the Residual Standard Deviation (RSD) of the linear regression in each of the two parts. A phenomenon of heteroscedasticity between the quasi-congested regime and the free-flow regime has been, here, measured. An important fact is that the model reproduces and maintains the same type of relation for all different days and for both regimes, as shown in Fig. 7. Computing the RSD of the linear regression of the data with an average speed $mi{n}_{T}\bar{u}(t)\ \le \ \bar{u}(t) < {u}^{* }$ with ${u}^{* }\in [7,13]$, we pointed out a drastic transition at ${u}_{qc}^{* }=11.4$ that determines a phenomenon of heteroscedasticity in the relation between $std({\bf{u}})$ and $\bar{u}$. In particular, ${u}_{qc}^{* }$ is the threshold that we take into account to define the quasi-congested regime and separate it from the free-flow condition. This important relation represents a global network behaviour that combines the topology structure with the traffic distribution and propagation.

## Discussion

The reaction-diffusion model had been applied in many different fields in science since its complete mathematical formulation. A common feature that all the previous applications shared was a physical and/or biological nature without neither external factors nor decision-makers. Applying such a system to traffic and congestion propagation might appear to exile the natural limits of the reaction-diffusion models. Nevertheless, we showed that the evolution of the speed values in a road network affected by daily congestion and the traffic emerging patterns can be accurately simulated by a reaction-diffusion differential system. We illustrated that it is possible to reproduce recurrent dynamics for the congestion propagation considering, on one hand, a general principle with a common reaction function, on the other hand, some diffusion and reaction parameters for the characteristics of the urban transportation system studied. An important observation is that the model parameters are defined based on principles of aggregated traffic models of MFD type. The ultimate scope of this work was to find how the traffic evolves given an initial state and a behavior for the global network speed.

We demonstrated that this kind of model is able to reproduce the average network link speeds with simple, global or regional, adaptive control parameters, the link speeds distribution and the congested spatial patterns during a whole day simulation. We obtained all these results without using any external information like traffic demand or drivers’ behavior that means not only light computational effort but also less real data needed.

Moreover, we pointed out the existence of an empirical relation between the network average speed and standard deviation of speed values distribution that appears in an urban network that experiences congestion and how this relation is reproduced by the reaction-diffusion model.

We believe that this work brings significant theoretical results and also computational advantages. In fact, in literature, it has been proved the existence of some internal and peculiar laws that connect the most common traffic variable like speed, density and flow, like the Macroscopic Fundamental Diagram Theory^19^ and also some similarities with physical systems^40,48,57^. We aimed to set a mathematical description of the congestion propagation which maintained the empirical relations in spatial networks with limited capacity, like the urban transportation systems.

Further applications of this work can be done for active traffic management and, in particular, for perimeter control as explained, for example, in refs. ^1,58,59^. In addition, because our model requires very few and aggregate data, like the average speed in a macro-region and a few parameters to be calibrated, it can be particularly adapted for the simulation of traffic in those cities in the developing countries where the cost of link detailed sensors remains not affordable.

### Data description

The data set used in this paper consists of several millions of daily taxis GPS data of the city of Shenzhen, China. Shenzhen is, nowadays, one of the most important cities in China, and the largest city in the Pearl River Delta region, in the south of Southern China’s Guangdong Province, known for a significant growth of its manufacturing market. In 1980, Shenzhen was designated China’s first Special Economic Zones (SEZs) and this testifies his crucial and important role in Chinese economic. The high and fast-growing density of people in Shenzhen, that in 2017 counted more than 12 millions of inhabitants, created, as expected, large congestion problems in the urban network. The daily dataset is composed by more than 40 millions of spatio-temporal GPS coordinates with a resolution of about 30 s for each taxi and each trip and they are located inside an area of about $140\ k{m}^{2}$ of the city center. In particular, we based our analysis on the estimated speed of 3 whole days, from ${7}^{th}$ to ${9}^{th}$ of September 2011, of the Shenzhen network profile. We showed the speed profile only for the ${7}^{th}$ (Day 1) as explanatory example. We used the other days for the calibration of the parameters $a$ and $b$ and to ascertain the stability of the model that maintains its accuracy for different traffic conditions. The estimation of average speed for each link has been done after applying a map matching algorithm in the trajectories of the vehicles. Then, the average space-mean speed every 5 minutes of those taxis that drove along that link, as the total distance travelled divided by the total time spent in the link during this period. In particular, the trajectories have been reconstructed by assigning each GPS point to the closest link on the road network and using the shortest path algorithm to select the links between two consecutive GPS traces. Then, the vehicular speed between two consecutive traces has been calculated dividing the partial trip length by the recorded time difference of the two GPS points. The network consists in $2013$ links and $1858$ points. To integrate the differential equation (1) we use an Euler integration with $dt=0.1$ and $T=14000$ time steps for a whole day simulation.

### Reaction-diffusion parameters ρ and σ. 

For the division of the traffic map of Shenzhen into clusters, we used the optimal solution of the algorithm described in ref. ^29^, the algorithm identifies 3 regions and, in particular, the green in Fig. 1 stands for the highway that surrounds the city center and the south and north part of the downtown that, for their different functional characteristics, may be consider separated and with slightly different parameters of the RD model for traffic propagation.

In general, $\rho$ and $\sigma$ will be represented by two squared matrices of dimension $R$, where $R$ is the number of the regions or types identified in the network. The calibration of these parameters has been done at looking at the best benchmark with the comparison with real data in terms of final average speed and distribution at regional level. In our case, with 3 regions obtained from the clustering algorithm described in ref. ^29^, we end up with the following values: $\rho =1{0}^{-2}\cdot (12,8,4;8,16,4;4,4,15)$ and $\sigma =1{0}^{-3}\cdot (1,0.5,0.2;0.5,1,0.2;0.2,0.2,1)$. The reasons at the bases of these values are the highest empirical correlation between roads belonging to the same clusters for traffic propagation dynamics. In particular, the third cluster (in green) delimited by the algorithms, highlights the highway that surrounds the city center and that it maintains a higher speed during the all day and, also it results less influenced by the neighbor links but of those adjacent and part of the same high-speed road (upstream and downstream links).

## Data Availability

All the data related to this study and its documentation are accessible using the following links: 10.6084/m9.figshare.7212230.
